# Limitations of CD44v6 amplification for the detection of tumour cells in the blood of colorectal cancer patients

**DOI:** 10.1054/bjoc.1999.1092

**Published:** 2000-03-06

**Authors:** D Masson, M G Denis, P Lustenberger

**Affiliations:** Laboratoire de Biochimie Spécialisée, Institut de Biologie, 9 quai Moncousu, 44035-Nantes, France

**Keywords:** CD44, exon v6, colorectal cancer, micrometastasis, reverse transcription polymerase chain reaction

## Abstract

Based on the important role of CD44 splice variants in colorectal cancer progression and metastasis, we evaluated the use of CD44v6 expression to detect and assess the metastatic potential of colorectal tumour cells circulating in peripheral blood. A nested amplification was designed that allowed to detect 10–100 colon cancer cells. This assay was applied to blood samples from healthy donors. Strong signals were detected in all cases, indicating that it cannot be used to detect colorectal carcinoma cells in whole blood. We then included an enrichment step based on the use of an anti-epithelial cells monoclonal antibody (BerEP4) coupled to magnetic beads. The CD44v6 reverse transcription polymerase chain reaction (RT PCR) assay was performed on cDNA synthesized from blood samples treated with these beads. We analysed 18 samples from 12 patients with a gastrointestinal disease, and 36 samples from ten patients with a colorectal cancer. None of the patients used as negative controls were found to contain epithelial cells in their blood as determined by cytokeratin 19 RT-PCR. By contrast, CD44 transcripts containing exon v6 were detected in nine out of the 18 samples tested (50%). For the colorectal cancer patients, six out of the seven samples (85.7%) that were cytokeratin 19-positive were CD44v6-negative, whereas ten samples out of the 29 not containing epithelial cells were CD44v6-positive (34.5%). This is probably due to the persistence of CD8+ leucocytes in the enriched preparations, as determined by PCR analysis of the CD8 α-chain. We conclude that detection of CD44v6 transcripts using a sensitive nested RT-PCR assay has no potential value to detect and characterize colorectal cancer micrometastases from blood, even following an initial enrichment step. © 2000 Cancer Research Campaign


British Journal of Cancer (2000) **82**(7), 1283–1289
© 2000 Cancer Research Campaign
DOI: 10.1054/ bjoc.1999.1092, available online at http://www.idealibrary.com on IDEAL®

### Limitations of CD44v6 amplification for the detection of tumour cells in the blood of colorectal cancer patients

**D Masson, MG Denis and P Lustenberger**

Laboratoire de Biochimie Spécialisée, Institut de Biologie, 9 quai Moncousu, 44035-Nantes, France

**Summary** Based on the important role of CD44 splice variants in colorectal cancer progression and metastasis, we evaluated the use of CD44v6 expression to detect and assess the metastatic potential of colorectal tumour cells circulating in peripheral blood. A nested amplification was designed that allowed to detect 10–100 colon cancer cells. This assay was applied to blood samples from healthy donors. Strong signals were detected in all cases, indicating that it cannot be used to detect colorectal carcinoma cells in whole blood. We then included an enrichment step based on the use of an anti-epithelial cells monoclonal antibody (BerEP4) coupled to magnetic beads. The CD44v6 reverse transcription polymerase chain reaction (RT PCR) assay was performed on cDNA synthesized from blood samples treated with these beads. We analysed 18 samples from 12 patients with a gastrointestinal disease, and 36 samples from ten patients with a colorectal cancer. None of the patients used as negative controls were found to contain epithelial cells in their blood as determined by cytokeratin 19 RT-PCR. By contrast, CD44 transcripts containing exon v6 were detected in nine out of the 18 samples tested (50%). For the colorectal cancer patients, six out of the seven samples (85.7%) that were cytokeratin 19-positive were CD44v6-negative, whereas ten samples out of the 29 not containing epithelial cells were CD44v6-positive (34.5%). This is probably due to the persistence of CD8+ leucocytes in the enriched preparations, as determined by PCR analysis of the CD8 α-chain. We conclude that detection of CD44v6 transcripts using a sensitive nested RT-PCR assay has no potential value to detect and characterize colorectal cancer micrometastases from blood, even following an initial enrichment step. © 2000 Cancer Research Campaign

**Keywords:** CD44; exon v6; colorectal cancer; micrometastasis; reverse transcription polymerase chain reaction

Metastasis is an important factor that regulates the prognosis of patients with cancer. Tumour cells are scattered from the original site, spreaded haematogenously and arrested at small vessels. These cells must then adhere to the vascular endothelium, migrate into the extracellular space and grow into secondary tumours. Detection of these tumour cells circulating in peripheral blood would be of interest to detect micrometastases at an early stage. This might result in a more appropriate selection of patients for adjuvant therapy.

A few tumour cells can be detected in a great excess of non-malignant cells using nucleic acid amplification by means of the polymerase chain reaction (PCR). In most cases, the detection relies on the tissue specific expression of a gene (for recent reviews see Denis et al, 1996; Pelkey et al, 1996; Verkaik et al, 1997; Bustin and Dorudi, 1998; Keilholz, 1998; Raj et al, 1998). This gene has to be expressed in cancer cells but not in cells that are normally present in blood. It can even be expressed in the corresponding normal tissue if these normal cells do not circulate in blood. Total RNA is extracted from whole blood or from nucleated cells. Messenger RNAs are reverse transcribed and the desired cDNA is amplified by PCR using specific oligonucleotides.

In the past few years, several reports have illustrated the application of this methodology to the detection of circulating colorectal micrometastases. In most cases, cytokeratins gene expression has been used to detect these epithelium-derived cells (Burchill et al, 1995; Funaki et al, 1997; Le Maire et al, 1998; Weitz et al, 1998; Wyld et al, 1998). Detections relying on the expression of the carcinoembryonic antigen gene (Castells et al, 1998; Ko et al, 1998) and of apolipoprotein AI (Normanno et al, 1998) have also been recently documented. We previously reported (Denis et al, 1997) the detection of tumour cell dissemination by reverse transcription polymerase chain reaction (RT-PCR) in a pilot study of patients undergoing surgery for colorectal cancer by using a combination of immunomagnetic separation and cytokeratin 19-nested RT-PCR.

In all these reports, the phenotype of the cells is used. One would like to assess the metastatic potential of these circulating cells. This would help to distinguish between a harmless circulating epithelial cell and one which would have given rise to a metastasis. Therefore, there is need for more specific markers.

Colorectal tumorigenesis is a complex multistep process in which genetic and epigenetic alterations accumulate to bring about the neoplastic phenotype (Vogelstein and Kinzler, 1993). These alterations provide putative markers for tumour progression. Amongst the epigenetic alterations, aberrant expression of variants of the CD44 gene (CD44v) is frequent and some isoforms might have an important role in tumour progression suggesting a complex role for CD44 in colorectal malignancy. Although contradictory findings have been reported in colorectal carcinoma, several groups have described a correlation between tumour progression and CD44v6 expression (Wielenga et al, 1993; Finn et al, 1994; Mulder et al, 1995). CD44v6 overexpression has

*Received 6 May 1999*
*Revised 13 October 1999*
*Accepted 1 November 1999*

*Correspondence to:* P Lustenberger recently been associated with an unfavourable prognosis in this disease (Mulder et al, 1994; Ropponen et al, 1998; Wielenga et al, 1998). We therefore decided to evaluate CD44v6 RT-PCR as a marker to assess the metastatic potential of circulating colorectal cancer cells in peripheral blood.

### MATERIALS AND METHODS

### Cell lines

HT29, SW480 and HCT8R colon carcinoma cell lines were grown in RPMI-1640 containing L-glutamine and sodium bicarbonate (Sigma, Saint Quentin Fallavier, France) supplemented with 10% fetal calf serum, penicillin (100 units $ml^{-1}$) and streptomycin (0.1 mg $ml^{-1}$). Cells were maintained at 37°C in 5% carbon dioxide and passaged twice a week.

### Blood samples and patients

Blood samples (5 ml) were collected in heparinized tubes and stored at 4°C for a maximum of 2 h before treatment. For colorectal cancer patients, blood sampling was performed immediately before the beginning of surgery. Staging was performed according to Astler and Coller (1954). Two stage B1, three stage B2, two stage C2 and three stage D patients were included in this study. The mean age of the patients (four women and six men) was 64 years (range 48–81, median 66).

Blood samples collected from patients with gastrointestinal diseases were used as controls. Twelve patients were studied: four gastric ulcers, three colitis, one sigmoiditis, one purpura, one Crohn's disease, one gastritis and one diverticulosis. The mean age of these patients (four women and eight men) was 61.4 years (range 17–88, median 68).

### RNA extraction

Total cellular RNA was extracted from cultured cells and normal peripheral blood nucleated cells with TRIZOL reagent (Life Technologies) according to the manufacturer's instructions.

### RT-PCR

The reverse transcription reaction and PCR amplification were performed in a Crocodile III thermal reactor (Appligene, Illkirch, France). Aerosol-resistant tips (CML, Nemours, France) were used to prevent contamination.

Total RNA was heated at 72°C for 3 min, and then cooled on ice. It was then combined with 0.5 μg of random hexamers (Promega, Lyon, France), transcription buffer (50 mM Tris–HCl pH 8.3, 75 mM potassium chloride (KCl), 3 mM magnesium chloride ($MgCl_2$), 10 mM dithrothreitol (DTT), dNTPs (1 mM each), RNAasin (50 units; Promega) and RNAaseH- M-MLV reverse transcriptase (200 units; Promega) in a total volume of 20 μl. Incubation was performed at 42°C for 60 min.

Amplifications were performed with 2 μl of cDNA in a total volume of 50 μl containing 1 × PCR buffer (10 mM Tris–HCl pH 9.0, 50 mM KCl, 1.5 mM $MgCl_2$, 0.1% Triton X-100 and 0.2 mg $ml^{-1}$ gelatin), 200 μM of each dNTP, 100 pmoles outer primers, and 2.5 units *Taq* DNA polymerase (Life Technologies). For reamplification with the nested primers, 0.5 μl of the first amplification was amplified in a final volume of 50 μl with inner primers as described above.

Cytokeratin 19 PCR were performed as previously described (Denis et al, 1997).

For CD44 amplifications, primers were designed from the published sequence for human CD44 gene (Screaton et al, 1992). Their location is presented on Figure 1. The sequence of these primers were:

| | |
|---|---|
| RC1 – 5′ primer: | 5′-AGCAACCCTACTGATGAT-GACG-3′ |
| v6.1 – 3′ primer: | 5′-GGAGTCTTCTCTGGGT-GTTTGG-3′ |
| RC2 – 5′ nested primer: | 5′-AGGAGGTTACATCTTTTA-CACC-3′ |
| v6.2 – 3′ nested primer: | 5′-ACCACTGTTCTTCTGGG-TAGC-3′ |

Thirty-five cycles were carried out (92°C for 30 s, 60°C for 30 s, 72°C for 60 s) followed by a 3 min final extension at 72°C.

Primers to amplify a fragment of the CD8 α-chain were designed from the published sequence (Littman et al, 1985) using the Genejockey™ software (Biosoft, Cambridge, UK). They were selected on different exons of the CD8 gene (Nakayama et al, 1989) so that DNA amplified from genomic DNA would be larger than the fragment amplified from cDNA. The sequence of these primers were:

| | |
|---|---|
| CD8/1 – 5′ primer: | 5′-GCGGTTCTCGGGCAA GAGGTT-3′ |
| CD8/2 – 3′ primer: | 5′-GCGATGGTGGGCGCCG GTGTT -3′ |
| CD8/3 – 5′ nested primer: | 5′-GTCCTCACCCTGAGCGA CTTC -3′ |
| CD8/4 – 3′ nested primer: | 5′-CGTCGTGTGGGCTTCGC-TGG -3′. |

For amplifications and nested amplifications the following thermal profile was used: 92°C for 20 s, 62°C for 30 s, 72°C for 20 s, for 35 cycles, followed by a 3 min final extension at 72°C. The expected size for the nested amplification was 126 bp.

### Gel electrophoresis of PCR products

RT-PCR products were analysed by electrophoresis on 2% agarose gels and visualised with ethidium bromide. A 100-bp DNA ladder (Promega) was used as size markers.

### Immunomagnetic separation

Blood samples were washed 3 times with cold phosphate-buffered saline (PBS) and 1-ml samples were transferred to separate tubes and analysed independently. Magnetic beads covalently coated with monoclonal antibody BerEP4 (DYNAL, Oslo, Norway) were then added ($4 \times 10^6$ beads $ml^{-1}$ of blood). Following incubation performed at 4°C for 30 min, cells bound to the beads were retrieved using a magnetic field. The beads were then washed 3 times with PBS. Total RNA was extracted from the cells immobilized on the beads with TRIZOL reagent. cDNA was synthesized and amplified as described above.

 © 2000 Cancer Research Campaign

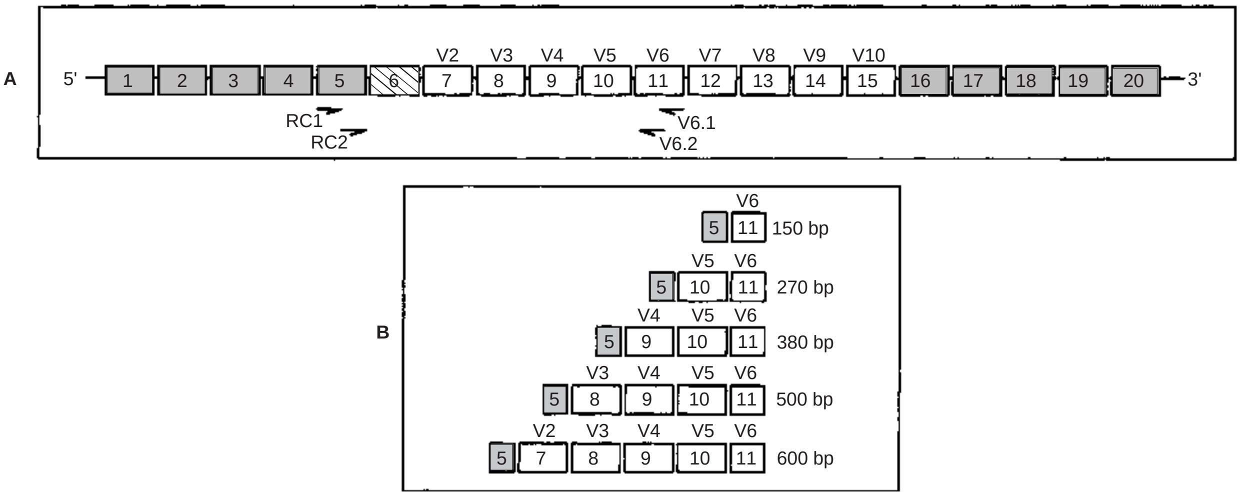

**Figure 1** (**A**) Schematic representation of the coding region of the human CD44 gene (dark boxes, constant exons; clear boxes, variant exons) and localization of the primers used to amplify CD44 transcripts containing exon v6. Exon 6 or v1 (stripped box) is not expressed in humans (Screaton et al, 1993). A first amplification was performed using oligonucleotides RC1 and v6.1 (PCR1). A nested PCR amplification was then performed with oligonucleotides RC2 and v6.2 (PCR2). (**B**) Schematic representation of the different CD44 splice transcripts detected in colon cancer cells (Figure 2)

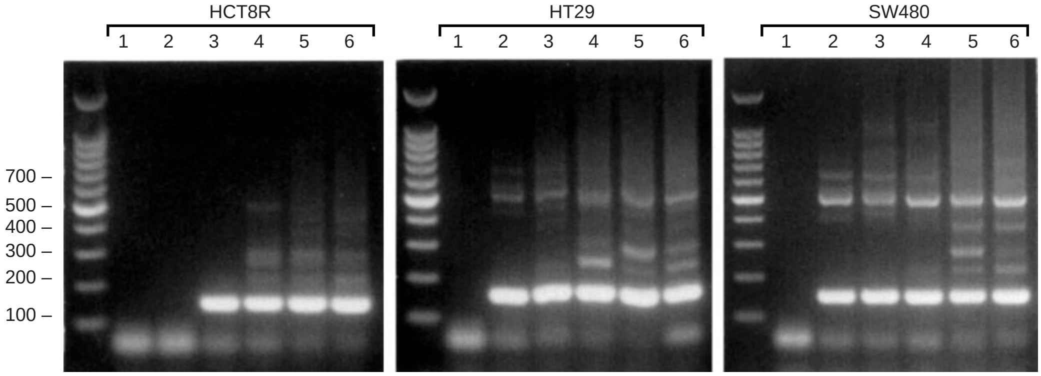

**Figure 2** Sensitivity of the nested RT-PCR. Total cellular RNA was extracted from HCT8R, HT29 and SW480 colon cancer cells. Reverse transcription and amplifications were performed as detailed in Materials and Methods from ten cells (2), 100 cells (3), 1000 cells (4), 10 000 cells (5), 100 000 cells (6) or water as negative control (1)

### RESULTS

### Sensitivity

We have used a combination of reverse transcriptase and polymerase chain reaction (two successive amplifications) to detect mRNA species containing exon 11 (v6) using 5′ primers specific for the constant region (exon 5) and 3′ primers specific for this variant exon (Figure 1A). We first determined the sensitivity of this assay. Total RNA was extracted from $10^5$ colorectal cancer cells. Serial dilution of RNA were then performed and processed for cDNA synthesis and amplification. A clear signal was obtained with RNA corresponding to ten HT29 or SW480 colon carcinoma cells, or 100 HCT8R cells (Figure 2). In all cases, the prominent fragment amplified was approximately 150-bp long. This corresponds to a transcript where no variant exon is present between the constant domain (exon 5) and the v6 exon (exon 11). Additional 270-bp, 380-bp, 500-bp and 600-bp amplicons were also detected. These fragments were purified from agarose gels and sequenced. This analysis revealed that they correspond to mRNA molecules in which, between exons 5 and 11, were inserted exon v5, exons v4–v5, v3–v4–v5 and v2–v3–v4–v5, respectively (Figure 1B). This pattern is in complete agreement with the variety of CD44 mRNAs described for colon cancer cell lines (van Weering et al, 1993).

### RT-PCR analysis of whole blood

We then analysed normal blood samples collected in heparinized tubes. Erythrocytes were lysed and total cellular RNA was extracted from the nucleated cells. Reverse transcription and amplification were performed. As shown in Figure 3, the nested amplifications yielded similar amplified products in all the normal samples analysed. As seen for colon cancer cell lines, different DNA fragments corresponding to different combinations of variant exons were amplified. These variants are expressed by normal cells present in the blood of healthy donors. Therefore, this amplification procedure cannot be used to detect colorectal carcinoma cells in whole blood.

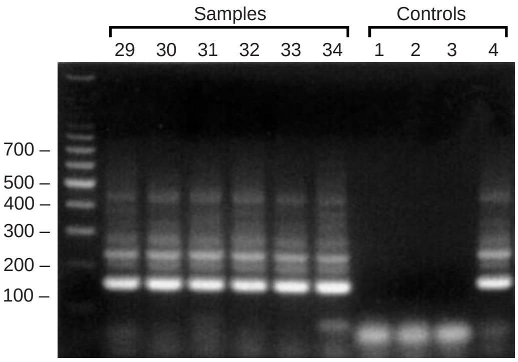

**Figure 3** Nested RT-PCR detection of CD44 transcripts containing exon v6 in RNA extracted from whole blood. Total cellular RNA was prepared from nucleated peripheral blood cells of six healthy volunteers (29–34). Reverse transcription and amplifications were performed as detailed in Materials and Methods. Negative controls were performed using water for the reverse transcription (1), the first PCR (2) and the nested amplification (3). A positive control was performed with RNA from HT29 colon cancer cells (4)

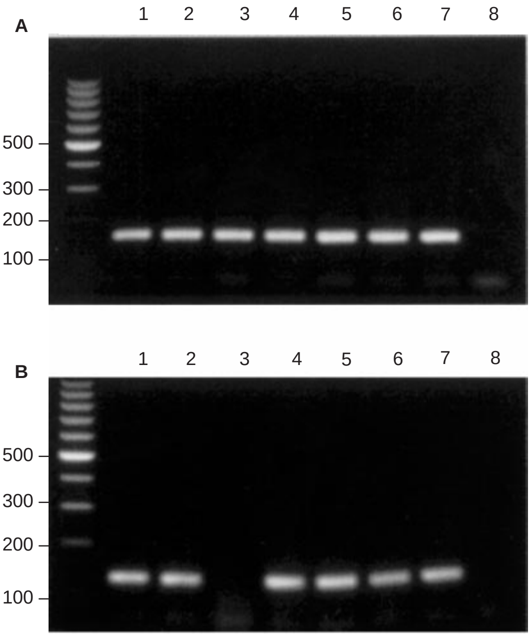

**Figure 4** Detection of CD44 RNA transcripts containing exon v6 following immunomagnetic separation. Blood samples from patients with a non-cancerous gastrointestinal disease (1–4) or from patients with a colorectal cancer (5–8) were treated with anti-epithelial cells magnetic beads. Reverse transcription and amplifications were performed as detailed in Materials and Methods. The cytokeratin 19 amplifications were all negative. The CD44v6 amplifications (**A**) and the CD8 α chain amplifications (**B**) are shown

**Table 1** RT-PCR analysis of samples from 12 patients with a gastrointestinal disease

| | Samples | PCR CD44v6+ | PCR CD44v6– |
|---|---|---|---|
| PCR cytokeratin 19+ | 0 | 0 | 0 |
| PCR cytokeratin 19– | 18 | 9 (50%) | 9 (50%) |

**Table 2** RT-PCR analysis of samples from ten colorectal cancer patients

| | Samples | PCR CD44v6+ | PCR CD44v6– |
|---|---|---|---|
| PCR cytokeratin 19+ | 7 | 1 (14.3%) | 6 (85.7%) |
| PCR cytokeratin 19– | 29 | 10 (34.5%) | 19 (65.5%) |

### RT-PCR following immunomagnetic separation

In order to get rid of most normal nucleated cells, we included an enrichment step based on the use of a specific monoclonal antibody coupled to magnetic beads. We used monoclonal antibody BerEP4 which recognizes an epitope on the protein moiety of two 34-kDa and 39-kDa glycopeptides expressed at the surface of epithelial cells in normal and malignant tissues (Momburg et al, 1987) and colorectal tumours (Latza et al, 1990). We have previously described the use of this reagent to isolate epithelial cells from blood samples (Denis et al, 1997). RNA is extracted from the cells immobilized on the magnetic beads. Nested RT-PCR is then performed using oligonucleotides designed from the sequence of the cytokeratin 19 gene. We are thus able to detect one epithelial cell in 1 ml of whole blood (Denis et al, 1997).

In the present report, we analysed 54 samples from 22 patients: 12 patients with a gastrointestinal disease (18 samples), used as controls, and ten patients with a colorectal cancer before they underwent surgery (36 samples). All these samples were first assayed for the presence of epithelial cells by cytokeratin 19 RT-PCR. We also applied the RT-PCR amplification using CD44 oligonucleotides to cDNA synthesized from blood samples treated with these magnetic beads.

None of the patients used as negative controls were found to contain epithelial cells in their blood (Table 1). By contrast, we were able to detect CD44 transcripts containing exon v6 in 9 out of these 18 samples tested (50%).

For the colorectal cancer patients, seven samples (from one stage B1, two stage B2 and three stage D patients) were found to contain epithelial cells (Table 2). Six out of the seven 1-ml samples that were cytokeratin 19-positive were CD44v6-negative. This is most likely related to the different sensitivity of the markers used for RT-PCR: one cell for cytokeratin 19, 10–100 cells for CD44v6. More importantly, ten samples out of the 29 samples which did not contain epithelial cells (34.5%) were found to be CD44v6-positive. These data were analysed by $\chi^2$ test. There was no association between the results of cytokeratin 19 and CD44v6 amplifications ($P$ = 0.29) for colorectal cancer patients.

We have not been able to demonstrate any significant quantitative or qualitative differences between amplified fragments containing CD44v6 from patients with non-cancerous gastrointestinal disease and patients with colorectal cancer. The major fragment amplified corresponded to the mRNA in which exon v6 is directly linked to the constant domain (150 bp, Figure 4A). Larger fragments were barely detectable. Finally, there was no statistically significant difference between the percentage of CD44v6 PCR-positive results obtained for the gastrointestinal diseases and the colorectal cancers ($\chi^2$ test, $P$ = 0.16).

In an attempt to identify cells of non-epithelial origin present in the enriched preparations that express CD44v6, we performed additional amplifications on these cDNAs. In a recent report, Wittig et al (1999) evaluated the expression of CD44 variant isoforms on peripheral blood leucocytes by flow cytometry. CD44v6 was predominant detected on CD8+ cells from healthy donors. Therefore, we performed amplifications with oligonucleotides derived from the sequence of the CD8 α-chain.

 © 2000 Cancer Research Campaign

Figure 4 illustrates the results obtained with eight samples (four from gastrointestinal disease patients and four from colorectal cancer patients) that did not contain epithelial cells (PCR for cytokeratin 19-negative). Seven of these eight samples were found to contain CD44v6-expressing cells (panel A). Six samples were positive for CD8 α-chain (Figure 4B). This clearly indicates that most of the preparations obtained following immunomagnetic separation still contain CD8+ lymphocytes. Sample 3 is negative for CD8 α-chain, but the preparation could contain other blood cell types, such as CD4+ or CD19+ lymphocytes, some of which also express CD44v6 (Wittig et al, 1999).

The conclusion, therefore, is that even if most nucleated cells normally present in blood are removed by the immunomagnetic separation, detection of CD44 transcripts containing exon v6 using a sensitive nested RT-PCR assay does not allow identification of circulating epithelial cancer cells.

### DISCUSSION

CD44 is a widely distributed cell surface adhesion molecule that is involved in critical biological processes such as lymphocyte homing and activation, cell motility, cell–matrix interaction and the regulation of tumour cell growth and metastasis (reviewed in Naot et al, 1997). Primary transcripts of the CD44 gene can be alternatively spliced to produce a variety of mRNA species (Screaton et al, 1992). The most abundant transcript corresponds to the standard or haematopoietic form. This RNA lacks the entire variable region, with exon 5 being linked to exon 16 (Figure 1). Alternatively spliced variants expressed in rat tumours have been shown to confer metastatic potential to non-metastatic carcinoma cell lines (Günther et al, 1991), and human homologues of rat variant mRNA sequences are expressed in human tumours (Heider et al, 1993).

For the detection of circulating epithelial-derived tumour cells in peripheral blood, the haematopoietic form is unsuitable because it is the most widely expressed type of CD44 in blood and in normal or tumoural colorectal tissue (Imazeki et al, 1996). Conversely, in patients with non-malignant disorders of the haematopoietic system, expression of CD44 variants in peripheral blood leucocytes was very low (Khaldoyanidi et al, 1996).

In normal colon mucosa, CD44v6 was present and detected on the colonic epithelium predominantly in the crypt base (Orzechowzki et al, 1995; Gotley et al, 1996; Givehchian et al, 1998). Standard and variant CD44 transcripts were both increased in carcinomas as compared with the corresponding normal mucosa (Gorham et al, 1996). Each variant exon from v2 to v8–v10 was detected in tumours for each Dukes' stage (Gotley et al, 1996). However, CD44v6 is the variant exon that has been most frequently associated with metastasis. In tumour tissues the abundant CD44v6 transcripts were localized exclusively in the cancer cells (Gorham et al, 1996). Abundant CD44v6 expression could be found in benign adenomatous polyps as well as in hepatic metastases of colorectal tumours (Heider et al, 1993). Several authors have reported that the increased expression of CD44v6 was a marker of tumour progression in human colorectal cancer (Wielenga et al, 1993; Mulder et al, 1995; Ropponen et al, 1998). Others have reported discordant results without any correlation between the expression or location of the CD44v6 protein and clinical parameters such as tumour grade, stage or patient survival (Gotley et al, 1996; Givehchian et al, 1998). The conclusion of these discordant studies were based on immunohistochemical analyses using different antibodies which might explain this discrepancy. Anyhow, CD44v6 is overexpressed during colorectal tumorigenesis in man and detected by immunochemistry in > 80% of tissue specimens from benign adenomatous polyp to colonic adenocarcinomas (Gotley et al, 1996). Expression of CD44v6 can thus be considered as an early event in colorectal carcinogenesis (Kim et al, 1994; Imazeki et al, 1996). Thus CD44v6 may be a particularly useful target for identification of circulating colorectal cancer cells.

We designed a highly sensitive nested RT-PCR assay based on 5′ oligonucleotides located on the constant region (exon 5), and 3′ oligonucleotides on the v6 exon (exon 10). The amplified products were thus of limited and well-defined size. Using this amplification method, the potential of CD44v6 expression to detect colorectal tumour cells in peripheral blood has been evaluated. In all mRNA preparations derived from normal blood, we identified a similar pattern of alternatively spliced CD44 transcripts. This nested amplification assay is more sensitive than the RT-PCR and Southern blot hybridization presented in their preliminary report by Wong et al (1997). A complex pattern of CD44 transcripts was amplified, suggesting that variant mRNAs are expressed by normal peripheral blood nucleated cells. During completion of this manuscript, similar observation were reported for exons 8–11 (Eltahir et al, 1998).

A fraction of blood mononucleated cells of healthy donors have been shown to express CD44v6 (Khaldoyanidi et al, 1996; Wittig et al, 1999). Alternative splicing of CD44 isoforms in human peripheral blood leucocytes is differentially regulated and depends on their stage of activation (Wittig et al, 1999). The major CD44 isoform on lymphocytes is the smallest standard molecule. T lymphocytes, upon activation up-regulate expression of specific CD44 variants (Koopman et al, 1993). A similar up-regulation of CD44 splice variants can be found upon activation of B lymphocytes (Kryworuckho et al, 1995). CD44v6 expression is associated with differentiation of monocytes to tissue macrophages in vivo in inflammatory sites (Levesque et al, 1996).

To increase the specificity of the assay, immunomagnetic beads, labelled with an epithelium-specific monoclonal antibody, were used to isolate epithelial colorectal tumour cells from blood and remove most of the CD44v6-expressing cells of non-epithelial origin. Unfortunately, the combination of these two techniques, one based on the detection of a membrane antigen by a monoclonal antibody, the other one based on nested RT-PCR, was still not specific as judged by the high rate of false-positives in our control group. We clearly demonstrated that CD8+ cells are still present in most of the samples following immunomagnetic separation. At the moment, we cannot exclude the presence of other subsets of blood cells in these preparations.

All cancer staging systems seek to identify clinical and pathological features that can predict outcome or guide therapy. Detection of epithelial tumour cells in blood might help in the selection of patients at high risk for metastases in the group of patients who are assessed presently with a good prognosis. Detection of early blood micrometastases in some of these patients might be used to guide therapy. We are at present investigating whether stage B and C colorectal cancer patients with occult potential micrometastases in their blood, detected by immunomagnetic separation and cytokeratin 19 RT-PCR, have a significantly decreased long-term survival or not. But there is still a need for markers that could be used to assess the metastatic potential of circulating cells.

© 2000 Cancer Research Campaign

### ACKNOWLEDGEMENTS

We wish to thank Prof. LeBorgne and Prof. LeHur (Chirurgie Générale II, Hôtel-Dieu, Nantes), Prof. LeNeel and Dr LeTessier (Clinique Chirurgicale A, Hôtel-Dieu, Nantes) and Prof. Galmiche (Gastroentérologie, Hôtel-Dieu, Nantes) for providing us with the blood specimen, and Dr Cassagnau for the anatomopathological data. This work was supported in part by the European Brite Mebioce project, and the Association pour la Recherche contre le Cancer (Grant No 5267).

### REFERENCES

Astler VB and Coller FA (1954) The prognostic significance of direct extension of carcinoma of the colon and rectum. *Ann Surg* **139**: 846–851

Burchill SA, Bradbury MF, Pittman K, Southgate J, Smith B and Selby P (1995) Detection of epithelial cancer cells in peripheral blood by reverse transcriptase-polymerase chain reaction. *Br J Cancer* **71**: 278–281

Bustin SA and Dorudi S (1998) Molecular assessment of tumour stage and disease recurrence using PCR-based assays. *Mol Med Today* **4**: 389–396

Castells A, Boix L, Bessa X, Gargallo L and Pique JM (1998) Detection of colonic cells in peripheral blood of colorectal cancer patients by means of reverse transcriptase and polymerase chain reaction. *Br J Cancer* **78**: 1368–1372

Denis MG, Truchaud A and Lustenberger P (1996) Detection de metastases de tumeurs solides dans la circulation sanguine par PCR. *Immunoanal Biol Spec* **11**: 361–371

Denis MG, Lipart C, Leborgne J, Lehur PA, Galmiche JP, Denis M, Ruud E, Truchaud A and Lustenberger P (1997) Detection of disseminated tumor cells in peripheral blood of colorectal cancer patients. *Int J Cancer* **74**: 540–544

Eltahir EM, Mallinson DS, Birnie GD, Hagan C, George WD and Purushotham AD (1998) Putative markers for the detection of breast carcinoma cells in blood. *Br J Cancer* **77**: 1203–1207

Finn L, Dougherty G, Finley G, Meisler A, Becich M and Cooper DL (1994) Alternative splicing of CD44 pre-mRNA in human colorectal tumors. *Biochem Biophys Res Com* **200**: 1015–1022

Funaki NO, Tanaka J, Itami A, Kasamatsu T, Ohshio G, Onodera H, Monden K, Okino T and Imamura M (1997) Detection of colorectal carcinoma cells in circulating peripheral blood by reverse transcription-polymerase chain reaction targeting cytokeratin-20 mRNA. *Life Sci* **60**: 643–652

Givehchian M, Worner S, Strater J, Zoller M, Heuschen U, Heuschen G, Lehnert T, Herfarth C and Von Knebel Doeberitz M (1998) No evidence for cancer-related CD44 splice variants in primary and metastatic colorectal cancer. *Eur J Cancer* **34**: 1099–1104

Gorham H, Sugino T, Woodman AC and Tarin D (1996) Cellular distribution of CD44 gene transcripts in colorectal carcinomas and in normal colonic mucosa. *J Clin Pathol* **49**: 482–488

Gotley DC, Fawcett J, Walsh MD, Reeder JA, Simmons DL and Antalis TM (1996) Alternatively spliced variants of the cell adhesion molecule CD44 and tumour progression in colorectal cancer. *Br J Cancer* **74**: 342–351

Gunthert U, Hofmann M, Rudy W, Reber S, Zoller M, Haussmann I, Matzku S, Wenzel A, Ponta H and Herrlich P (1991) A new variant of glycoprotein CD44 confers metastatic potential to rat carcinoma cells. *Cell* **65**: 13–24

Heider KH, Hofmann M, Hors E, Van Den Berg F, Ponta H, Herrlich P and Pals ST (1993) A human homologue of the rat metastasis-associated variant of CD44 is expressed in colorectal carcinomas and adenomatous polyps. *J Cell Biol* **120**: 227–233

Imazeki F, Yokosuka O, Yamaguchi T, Ohto M, Isono K and Omata M (1996) Expression of variant CD44-messenger RNA in colorectal adenocarcinomas and adenomatous polyps in humans. *Gastroenterology* **110**: 362–368

Khaldoyanidi S, Achtnich M, Hehlmann R and Zoller M (1996) Expression of CD44 variant isoforms in peripheral blood leukocytes in malignant lymphoma and leukemia: inverse correlation between expression and tumor progression. *Leuk Res* **20**: 839–851

Keilholz U (1998) New prognostic factors in melanoma: mRNA tumour markers. *Eur J Cancer* **34**: 37–41

Kim H, Yang XL, Rosada C, Hamilton SR and August JT (1994) CD44 expression in colorectal adenomas is an early event occurring prior to K-ras and p53 gene mutation. *Arch Biochem Biophys* **310**: 504–507

Ko Y, Klinz M, Totzke G, Gouni-Berthold I, Sachinidis A and Vetter H (1998) Limitations of the reverse transcription-polymerase chain reaction method for the detection of carcinoembryonic antigen-positive tumor cells in peripheral blood. *Clin Cancer Res* **4**: 2141–2146

Koopman G, Heider KH, Horst E, Adolf GR, Van Den Berg F, Ponta H, Herrlich P and Pals ST (1993) Activated human lymphocytes and aggressive non-Hodgkin's lymphomas express a homologue of the rat metastasis-associated variant of CD44. *J Exp Med* **177**: 897–904

Kryworuckho M, Diaz-Mitoma F and Kumar A (1995) CD44 isoforms containing exons V6 and V7 are differentially expressed on mitogenically stimulated normal and Epstein–Barr virus-transformed human B cells. *Immunology* **86**: 41–48

Latza U, Niedobitek G, Schwarting R, Nekarda H and Stein H (1990) Ber-EP4: new monoclonal antibody which distinguishes epithelia from mesothelia. *J Clin Pathol* **43**: 213–219

Le Maire V, Sales JP, Wind P, Dumas F, Landi B, Fayemendy L, Cugnenc PH, Gayral F, Lacour B and Loric S (1998) Evaluation and interest in new molecular markers in colon cancer. *Ann Pharm Fr* **56**: 9–17

Levesque MC and Haynes BF (1996) In vitro culture of human peripheral blood monocytes induces hyaluronan binding and up-regulates monocyte variant CD44 isoform expression. *J Immunol* **156**: 1557–1565

Littman DR, Thomas Y, Maddon PJ, Chess L and Axel R (1985) The isolation and sequence of the gene encoding T8: a molecule defining functional classes of T lymphocytes. *Cell* **40**: 237–246

Momburg F, Moldenhauer G, Hämmerling GJ and Möller P (1987) Immunohistochemical study of the expression of a *M*r 34 000 human epithelium-specific surface glycoprotein in normal and malignant tissues. *Cancer Res* **47**: 2883–2891

Mulder JW, Kruyt PM, Sewnath M, Oosting J, Seldenrijk CA, Weidema WF, Offerhaus GJ and Pals ST (1994) Colorectal cancer prognosis and expression of exon-v6-containing CD44 proteins. *Lancet* **344**: 1470–1472

Mulder JW, Wielenga VJ, Polak MM, Van Den Berg FM, Adolf GR, Herrlich P, Pals ST and Offerhaus GJ (1995) Expression of mutant p53 protein and CD44 variant proteins in colorectal tumorigenesis. *Gut* **36**: 76–80

Nakayama K, Tokito S, Okumura K and Nakauchi H (1989) Structure and expression of the gene encoding CD8 alpha chain (Leu-2/T8). *Immunogenetics* **30**: 393–397

Naot D, Sionov RV and Ish-Shalom D (1997) CD44: structure, function, and association with the malignant process. *Adv Cancer Res* **71**: 241–319

Normanno N, de Luca A, Castaldo A, Casamassimi A, Di Popolo A, Zarrilli R, Porcellini A, Acquaviva AM, Avvedimento VE and Pignata S (1998) Apolipoprotein A-I reverse transcriptase-polymerase chain reaction analysis for detection of hematogenous colon cancer dissemination. *Int J Oncol* **13**: 443–447

Orzechowski HD, Beckenbach C, Herbst H, Stolzel U, Riecken EO and Stallmach A (1995) Expression of CD44v6 is associated with cellular dysplasia in colorectal epithelial cells. *Eur J Cancer* **31A**: 2073–2079

Pelkey TJ, Frierson HF and Bruns DE (1996) Molecular and immunological detection of circulating tumor cells and micrometastases from solid tumors. *Clin Chem* **42**: 1369–1381

Raj GV, Moreno JG and Gomella LG (1998) Utilization of polymerase chain reaction technology in the detection of solid tumors. *Cancer* **82**: 1419–1442

Ropponen KM, Eskelinen MJ, Lipponen PK, Alhava E and Kosma VM (1998) Expression of CD44 and variant proteins in human colorectal cancer and its relevance for prognosis. *Scand J Gastroenterol* **33**: 301–309

Screaton GR, Bell MV, Jackson DG, Cornelis FB, Gerth U and Bell JI (1992) Genomic structure of DNA encoding the lymphocyte homing receptor CD44 reveals at least 12 alternatively spliced exons. *Proc Natl Acad Sci USA* **89**: 12160–12164

Screaton GR, Bell MV, Bell JI and Jackson DG (1993) The identification of a new alternative exon with highly restricted tissue expression in transcripts encoding the mouse Pgp-1 (CD44) homing receptor. Comparison of all 10 variable exons between mouse, human, and rat. *J Biol Chem* **268**: 12235–12238

Verkaik NS, Schroder FH and Romijn JC (1997) Clinical usefulness of RT-PCR detection of hematogenous prostate cancer spread. *Urol Res* **25**: 373–384

Vogelstein B and Kinzler KW (1993) The multistep nature of cancer. *Trends Genet* **9**: 138–141

van Weering DH, Baas PD and Bos JL (1993) A PCR-based method for the analysis of human CD44 splice products. *Pcr Methods Appl* **3**: 100–106

Weitz J, Kienle P, Lacroix J, Willeke F, Benner A, Lehnert T, Herfarth C and Von Knebel Doeberitz M (1998) Dissemination of tumor cells in patients undergoing surgery for colorectal cancer. *Clin Cancer Res* **4**: 343–348

Wielenga VJ, Heider KH, Offerhaus GJ, Adolf GR, Van Den Berg FM, Ponta H, Herrlich P and Pals ST (1993) Expression of CD44 variant proteins in human colorectal cancer is related to tumor progression. *Cancer Res* **53**: 4754–4756

Wielenga VJ, Van Der Voort R, Mulder JW, Kruyt PM, Weidema WF, Oosting J, Seldenrijk CA, Van Krimpen C, Offerhaus GJ and Pals ST (1998) CD44 splice variants as prognostic markers in colorectal cancer. *Scand J Gastroenterol* **33**: 82–87

 *© 2000 Cancer Research Campaign*

Wittig B, Seiter S, Schmidt DS, Zuber M, Neurath M and Zoller M (1999) CD44 variant isoforms on blood leukocytes in chronic inflammatory bowel disease and other systemic autoimmune diseases. *Lab Invest* **79**: 747–759
Wong LS, Cantrill JE, Morris AG and Fraser IA (1997) Expression of CD44 splice variants in colorectal cancer. *Br J Surg* **84**: 363–367
Wyld DK, Selby P, Perren TJ, Jonas SK, Allen-Mersh TG, Wheeldon J and Burchill SA (1998) Detection of colorectal cancer cells in peripheral blood by reverse-transcriptase polymerase chain reaction for cytokeratin 20. *Int J Cancer* **79**: 288–293

© 2000 Cancer Research Campaign
